# 

**Published:** 1856-05

**Authors:** 


### CONTENTS.

### ORIGINAL COMMUNICATIONS.

PAGE

ARTICLE I.—An Essay on Quackery, read before the Atlanta Medical Society, and published by that body. By T. C. H. WILSON, M. D., Atlanta, Ga. 513
ARTICLE II.—Reports from the Case Book of THOS. S. POWELL, M. D., Sparta, Georgia,........ 516
ARTICLE III.—Abnormal Placenta. By DR. J. R. JOHNSON, Lebanon, Ala. 518
ARTICLE IV.—Peculiarities and diversified symptoms manifested in Hysterical subjects. By J. G. WESTMORELAND, M. D., Atlanta, Georgia,........ 520
ARTICLE V.—Reports from the Records of the Atlanta Medical Society. By EBEN HILLYER, M. D., Secretary. April 3d, 1856,........ 524

**SELECTIONS.**

Progress of Discovery in the Nervous System and in Medical Ethics,........ 529
Considerations on the Reciprocal Influence of the Physical Organization and Mental Manifestations. By A. O. KELLOGG, M. D., Port Hope, Canada West,........ 533
Treatment of Croup,........ 541
Tuberculosis. By HENRY GOADBY, M. D., F. L. S........ 553
Case of Encysted Sanguineous Tumor of the Neck, successfully removed. By J. M. CARNOCHAN, M. D., Surgeon-in-Chief to the State Hospital, Professor of Surgery in the New York Medical College, etc.,........ 557

**EDITORIAL AND MISCELLANEOUS.**

The Second Session,........ 562
State Medical Society,........ 564
Museum, Atlanta Medical Men, &c........ 565
Publications received,........ 557
On the presentation of Dead Children,........ 574
Death of a Fœtus from Smallpox,........ 575
Medical Affairs in Europe,........ 575
Legal Responsibility,........ 576
Subscriptions received,........ 576

